# A Case of Cardboard Boxes Likely Facilitating the Biting of a Patient by *Trypanosoma cruzi*-Infected Triatomine Bugs

**DOI:** 10.4269/ajtmh.16-0455

**Published:** 2016-11-02

**Authors:** Eduardo P. Dolhun, Andrew W. Antes

**Affiliations:** 1 Department of Medicine, Stanford University, Stanford, California; 2 Dolhun Clinic, San Francisco, California

## Abstract

Chagas disease is a vector-borne and potentially fatal parasitic disease that is transmitted by the triatomine bug, a nocturnal feeding, flying arthropod, often referred to by its colloquial name, the “kissing bug.” Vector-borne transmission is considered the most important means of spreading Chagas disease in endemic and nonendemic areas. Corrugated cardboard boxes may accelerate the spread of these insect vectors to nonendemic areas through their ability to harbor and transport small terrestrial arthropods such as silverfish, termites, and cockroaches. We report the case of a patient living in northern California who presented to a community clinic 6 weeks after being bitten by a positively identified triatomine bug. A local pest control company identified a total of eight adult *Triatoma protracta*, nine nymphs, and two eggs; all within the patient's bedding. No bugs were found outside of the patient's bedroom. The Centers for Disease Control and Prevention confirmed one adult female was positive for *Trypanosoma cruzi* via polymerase chain reaction. The patient's bedroom doubled as an office and regularly received and stored corrugated cardboard shipping boxes. Corrugated cardboard boxes have been used to trap and study the triatomine bug. This is the first documented case that provides circumstantial evidence that corrugated cardboard boxes may be an inadvertent and unrecognized factor in the spread of Chagas disease.

## Case Report

A 26-year-old, previously healthy, professional male presented with a 6-week history of being bitten throughout his body. The patient lived with his parents in a well-manicured suburban home at the San Francisco Bay Area with no pets, and had not traveled to any countries known for high burden of Chagas disease (e.g., Latin America).

He originally self-treated bites on his left leg which were large, swollen, and red with Neosporin^®^ ointment (Johnson & Johnson, New Brunswick, NJ; bacitracin, neomycin, and polymyxin B) and over-the-counter hydrocortisone ointment. A few days later he visited his former pediatrician who prescribed sulfamethoxazole–trimethoprim (800/160 mg) twice daily, along with naproxen 500 mg twice daily.

He then left and traveled for work about 3 weeks. On return and sleeping in his own bed, he acquired eight new bites. At that point the patient stopped sleeping in his bed, wore new clothes, and drove a different car to work in an attempt to avoid new bites.

Thirty days after the original bites, he contacted a local pest control company (name withheld to maintain patient privacy) that inspected his car and bedroom, with the help of a bedbug-sniffing dog. They isolated eight adult reduviid bugs, nine nymphs, and two eggs residing in his bed sheets. Upon recommendation of the first pest company, a second pest company was engaged and confirmed that the insects were reduviid bugs.

Patient next contacted the Entomology Department at the Centers for Disease Control and Prevention (CDC) that identified the kissing bugs as *Triatoma protracta* based on five photos taken by the second pest control company. The patient then returned to his former pediatrician who obtained blood samples and ordered a radioimmunoprecipitation assay test from Quest Diagnostics^®^ (San Francisco, CA). He then sent three of the captured *T. protracta* bugs to the CDC. After 2 weeks, the CDC identified one of the samples as an adult female, assigned it a unique number, and confirmed that it had tested positive for *Trypanosoma cruzi*.

One and a half months after exposure, blood tests revealed a normal complete blood count. The metabolic panel was significant only for an elevated alanine aminotransferase (ALT). Repeat testing demonstrated a decrease in ALT 2 months later and was thought to be due to steatohepatitis. Giemsa stains for the *T. cruzi* parasites were negative, as was Chagas medium liver digest–neutralized tryptose testing.

The patient's bedroom doubled as his office, where he regularly kept shipping boxes. He estimated that he received eight boxes of various sizes per month from numerous online and brick and mortar stores, keeping four boxes within a few feet of his bed at any given time. Boxes were of domestic origin only and contained general office supplies and personal goods, such as clothing.

He was questioned, via text message, if he kept a relatively neat bedroom. Doctor: “Would you consider that your bedroom is tidy?” Patient: “My mom … is generally insanely (clean). The boxes were there because she didn't want them cluttering the house elsewhere. Other than boxes, room is exceptionally clean.”

Since removal of the Chagas bugs and boxes, there have been no reported bites to the patient, family members, or immediate neighbors after 2 years.

## Discussion

Chagas disease is a vector-borne and potentially fatal disease with growing incidence in the United States.^1^ The causative agent of this disease is a protozoan parasite, *T. cruzi*, which is traditionally transmitted through kissing bug feces contact with bite wounds, intact mucus membranes or conjunctiva, vertical or congenital transmission, blood transfusions, bone marrow or organ transplants, certain foods and drinks, or accidental laboratory exposure.^2^ Contact with infected feces of the arthropods belonging to one of 11 subspecies of the subfamily Triatominae, which belongs to the family Reduviidae, is the most common form of diseaseo transmission.^2^–^4^

**Figure 1.**
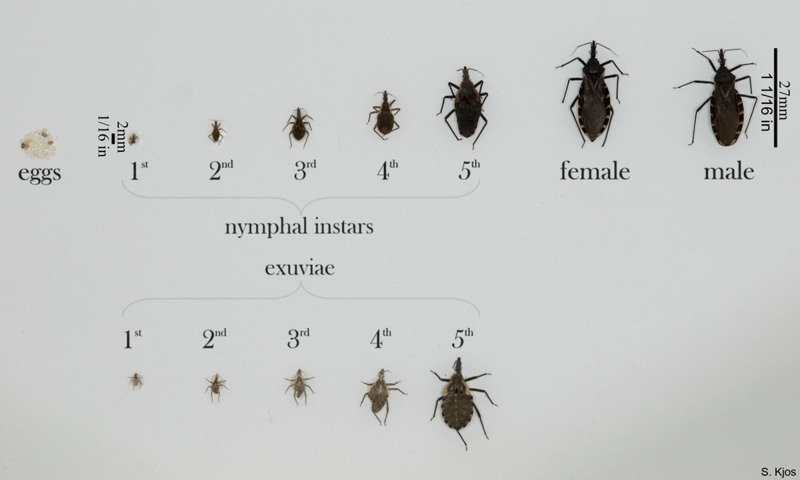
Various triatomine bugs in all life stages. CDC, 2010 (http://www.cdc.gov/parasites/chagas/gen_info/vectors/triatomine_stages_lg.html).

Reduviid bugs are known to reside in thatched roofs, unkempt bedrooms, and corrugated cardboard boxes,^5^ which have been used to trap these bugs for scientific purposes.^6^,^7^ These bugs lay their eggs, each measuring around 1 mm in diameter, near their established food source.^8^ Once hatched, nymphs go through five growth stages, known as instars, over the course of 1–2 years before becoming an adult insect, measuring up to 27 mm in length and 3.25–5.8 mm wide (see Figure 1

). Emerging from their hiding places each night, these flying, nocturnal, hematophagous insects feed on nearby sleeping hosts, earning the nicknames “kissing bug,” “assassin bug,” and “vampire bug.” Although not all kissing bugs carry *T. cruzi*, they may become infected after feeding on any of the 180 different species residing in the United States that have been documented as host reservoirs, including common animals like dogs, chickens, and rats.^9^,^10^

It is estimated that as many as 8–10 million people in Mexico, Central America, and South America have Chagas disease, most of them do not know they are infected. If untreated, infection is lifelong and can be life threatening.^11^,^12^ Chagas is recognized as the most economically important parasitic disease in Latin America.^1^

Historically considered nonendemic to California, the first indigenously acquired acute Chagas case involving a 56-year-old woman in Lake Don Pedro, CA, was described in 1985. The authors also reported that six of 10 dogs and 19 ground squirrels in her vicinity had been infected, and 0.2% of blood donors in the San Francisco Bay Area tested positive for *T. cruzi* antibodies.^13^ Kissing bugs have been documented in 27 states.^14^ In 2013, Texas became the fourth state to list Chagas as a reportable disease, joining Arizona, Tennessee, and Massachusetts.^15^ Given current trends in the spread of this disease, greater awareness and early intervention may prevent not only the spread of the disease, but, more importantly, the progression from acute to the potentially fatal and economically costly chronic phase of the disease.

Corrugated cardboard shipping boxes are ubiquitous. Fed Ex and the United States Postal Service alone deliver 6.5 billion packages per year in the United States.^16^ Of Amazon's 78 American fulfilment centers, which ship globally, 53 are located in states where the CDC has confirmed the presence of triatomine bugs.^14^ Depending on the intended use, corrugation sizes in standard cardboard boxes can range from 0.8 to 2.2 mm wide, and 0.03 to 5 mm high.^17^ Adult triatomes may seek out shelter within larger corrugations or small tears or defects of a box, whereas the younger, soft-bodied nymphs and eggs themselves may be able to reside within the smallest of corrugations found in commercial shipping and storage boxes, as has been observed in several other insect species.^18^,^19^

The fact that these nocturnal, bloodsucking insects tend to nest close to their host(s), repeatedly biting over the course of their relatively long lifetime, may be facilitated by sleeping in close proximity to cardboard boxes. This is the first documented case that provides circumstantial evidence that corrugated cardboard used for shipping, moving, and/or storage, may contribute to the spread of Chagas disease.
